# Antimicrobial Drug Resistance among Refugees from Syria, Jordan

**DOI:** 10.3201/eid2305.170117

**Published:** 2017-05

**Authors:** Aula Abbara, Nizar Al-Harbat, Nabil Karah, Bashar Abo-Yahya, Wael El-Amin, James Hatcher, Omar Gabbar

**Affiliations:** Imperial College, London, UK (A. Abbara);; Syrian American Medical Society, Canfield, Ohio, USA (A. Abbara);; Al Maqased Charity Hospital, Amman, Jordan (N. Al-Harbat, B. Abo-Yahya);; Umea University, Umea, Sweden (N. Karah);; Broomfield Hospital, Chelmsford, Essex, UK (W. El-Amin);; Imperial College Healthcare National Health Service Trust, London (J. Hatcher);; University Hospital Leicester, Leicester, UK (O. Gabbar)

**Keywords:** antimicrobial resistance, Syrian refugees, Jordan, Syria, multidrug resistant, bacteria, *Proteus*, *Escherichia coli*, *Pseudomonas*, *Klebsiella*, injury

**To the Editor:** The Kassem et al. article regarding high rates of multidrug-resistant (MDR) bacteria colonizing Syrian children highlights the challenge of choosing empiric antimicrobial drugs to treat war-injured refugees from Syria (*1*). The findings mirror other reports (*2**–**3*) and our own experience in a charitable hospital in Amman, Jordan, which manages war-injured refugees from Syria. As part of a program of antimicrobial drug stewardship and infection prevention and control, empiric antimicrobial drug protocols were introduced. For antimicrobial drug–naive patients, the first-line choice for prophylaxis and treatment of skin and soft-tissue infections, including those involving open fractures, was a narrow-spectrum cephalosporin, as recommended by the Infectious Diseases Society of America guidelines (*4*); however, clinical failure was common.

We retrospectively reviewed the clinical microbiology data of 75 patients admitted in January 2015 with a history of suspected post-trauma infection. All these patients were first treated in field hospitals in Syria; 82.7% were male, and 33% were <16 years old. Twenty-four percent had multiple injuries, 20% had osteomyelitis, and 53% had metal prosthetic implants.

Thirty bacterial isolates were identified, mostly from deep wound swabs of 21 (28%) injured patients; 9/21 were infected with 2 isolates. Twenty-nine (97%) isolates were gram-negative bacteria: 10 *Proteus* spp., 10 *Escherichia coli*, 5 *Pseudomonas* spp., and 4 *Klebsiella* spp. Disk diffusion susceptibility testing showed that 20 (66%) isolates were MDR and 11 (36.7%) were carbapenem resistant. 

The hospital laboratory did not have the capacity to perform further testing and confirmation of the resistant strains in line with international quality standards because they lacked suitable equipment and financial resources. Preventing further dissemination of MDR organisms among war-injured refugees from Syria at hosting healthcare facilities requires an effective surveillance system, investment in infection prevention and control, appropriate antimicrobial drug stewardship, and urgent laboratory capacity building inside Syria and in the refugee-host countries.
